# High-resolution physicochemical characterization of different intravenous immunoglobulin products

**DOI:** 10.1371/journal.pone.0181251

**Published:** 2017-07-31

**Authors:** Nathaniel Washburn, Robin Meccariello, Shaohui Hu, Maurice Hains, Naveen Bhatnagar, Hetal Sarvaiya, Bulbul Kapoor, John Schaeck, Ignacio Pino, Anthony Manning, Jonathan C. Lansing, Carlos J. Bosques

**Affiliations:** 1 Research, Momenta Pharmaceuticals, Cambridge, Massachusetts, United States of America; 2 Research, CDI Laboratories, Baltimore, Maryland, United States of America; 3 Research, CDI Laboratories, Mayaguez, Puerto Rico; Università degli Studi di Milano, ITALY

## Abstract

Intravenous immunoglobulin (IVIg) is a complex mixture drug comprising diverse immunoglobulins and non–IgG proteins purified from the plasma of thousands of healthy donors. Approved IVIg products on the market differ regarding source of plasma, isolation process, and formulation. These products are used widely, and often interchangeably, for the treatment of immunodeficiency and autoimmune and inflammatory diseases, but their mechanisms of action in different indications are not well understood. A primary limitation to understanding the therapeutic relevance of specific components within IVIg has been the limited resolution of analytics historically implemented to characterize its complex mixture. In this study, high-resolution analytics were applied to better understand the composition of IVIg and product variations. We characterized three approved IVIg products: Gammagard^®^, Privigen^®^, and Octagam^®^. Differences in the distribution of molecular weight species, IgG sequence variants, isoforms, glycoforms, and the repertoire of previously reported antibody specificities were identified. We also compared the effect of aging on these products to identify changes in size distribution and posttranslational modifications. This type of characterization may provide insights into the specific factors and components of IVIg that may influence its activity and ultimately lead to optimization of IVIg products for use in autoimmune diseases.

## Introduction

Intravenous immunoglobulin (IVIg) is a complex heterogeneous mixture of human antibodies (primarily polyclonal IgG) and non-IgG proteins that is indicated for use as immune replacement therapy in patients with primary and secondary immune deficiencies, and as an immunomodulatory treatment for a wide variety of autoimmune and inflammatory diseases [1,2]. IVIg is approved for primary humoral immunodeficiency, immune thrombocytopenic purpura, and multifocal motor neuropathy in the United States [3–6] and European Union [7–10] and for B-cell chronic lymphocytic leukemia (for hypogammaglobulinemia and recurrent bacterial infections), chronic inflammatory demyelinating polyneuropathy, and Kawasaki syndrome in the European Union [7–10]. IVIg is obtained from a large pool of healthy blood donors to ensure a diverse immunoglobulin repertoire [2], and the quality of IVIg lots are tested based on specifications set around IgG content (>95%) and size distribution (≥90% dimer + monomer and ≤3% aggregates/high molecular weight [MW] proteins) [11]. To ensure quality, IVIg lots are also routinely checked for viral clearance and endotoxin levels, and additional specifications are set around the levels of other non-IgG proteins, such as prekallikrein activator, IgA, and hemagglutinin titers [11].

Although specifications have been established to ensure the quality and safety of IVIg preparations [12], the complex composition of IVIg can, in principle, be influenced by manufacturing/purification processes and the donor pools that are the source of plasma used to generate IVIg, resulting in variations among manufacturers in the composition of IVIg preparations [11]. In this article we report the results from the analysis of different IVIg products for components that may have function in indications for autoimmune and inflammatory diseases. The following components were analyzed: MW distribution, IgG isotypes and allotypes, backbone modifications, IgG-Fc and fragment antigen binding (Fab) glycosylation, antigen binding repertoire, and non-IgG proteins. In addition to evaluating relative differences in composition between different IVIg products for these factors, we have also evaluated how they change as a function of time.

One of the proposed mechanisms of action of IVIg in inflammatory diseases is the modulation of Fc_γ_-receptors [13]. The distribution of IgG heavy chain isotypes may influence the activity of the mixture because it is known that each IgG isotype interacts differently with the different Fc_γ_-receptors [14]. For example, IgG1 and IgG3 isotypes bind certain Fc_γ_-receptors with higher affinity than IgG2 and IgG4 isotypes [14]; this in turn leads to more efficient effector function for IgG1 and IgG3 isotypes [15]. The four IgG isotypes can be further classified based on polymorphisms within the isotype, known as allotypes, are reflective of the population from which the plasma is drawn [16].

Furthermore, IgG4 isotypes have unique properties that may further influence the activity of IVIg, including its ability to form stable half antibodies (heavy chain–light chain pairs) that result in dynamic Fab arm exchange [17]. This Fab arm exchange can lead to the generation of bispecific IgG4 antibodies, which have been shown to have anti-inflammatory properties in animal models [17]. Furthermore, the presence of low abundance IgG4 isotypes in IVIg that are reactive against thyroglobulin (i.e., antithyroglobulin IgG4 antibodies) has been proposed to play an important therapeutic role in the activity of IVIg in Hashimoto’s disease, an autoimmune disorder [18].

IgG glycosylation is also thought to influence the activity of IVIg [19]. For example, Fc sialylation of IgG has been shown to play an important role in the anti-inflammatory activity of IVIg [20–22]. Terminal galactosylation has also been proposed to play a role in the activity of IVIg mediating the interaction with Dectin-1 [23]. Terminal galactose was also shown to decrease the affinity of the interaction of IgG with mannose-binding lectin in vitro [24]. The pairing of N-glycans in the Fc domain may also be important for activity, as shown in studies examining the binding of asymmetrically glycosylated IgG to Fc_γ_ receptors [25]. Additionally, some antibodies are glycosylated in the Fab domain, typically in the complementarity-determining regions. The glycans in the Fab domain can affect the affinity and avidity of anticitrullinated peptide antibodies [26]. Given the unknown impact of different IVIg components, our intent was to determine if applying high-resolution analytics could provide new insights into the complexity of IVIg therapy.

## Materials and methods

In this study, we characterized IVIg from three different manufacturers: Gammagard 10% (Baxter Healthcare, Westlake Village, CA) [6], Privigen 10% (CSL Behring AG, Bern, Switzerland) [5], and Octagam 5% (Octapharma USA Inc, Hoboken, NJ) [4] (Table 1). Three lots of IVIg drug product were obtained from each manufacturer and stored according to the manufacturer’s instructions from the time of receipt to the time of analysis. A single lot of each product was analyzed after the expiration date, as noted in Table 1.

**Table 1:** Intravenous immunoglobulin product information.

Product	Notation	Lot number	Expiration date relative to analysis date
Privigen	Pri-1	4323300094	22 months after
	Pri-2	4323300074	2 months prior
	Pri-3	4323300106	28 months after
Octagam	Oct-1	A150A8431	5 months prior
	Oct-2	A317C8431	12 months after
	Oct-3	A405E8431	24 months after
Gammagard	Gam-1	LE12M005AB	6 months after
	Gam-2	LE12GA97AB	36 months prior
	Gam-3	LE12M209AB	24 months after

SEC analysis was performed after diluting the drug product to 10 mg/mL in PBS (Life Technology, Carlsbad, CA, USA). 10 μL of each sample, equivalent to 100 μg, was injected on a Waters Alliance 2690 separations module (Waters Corporation, Milford, MA, USA) for separation on a Sepax Zenix-C HP-SEC column (Sepax Technologies, Newark, DE, USA) in a mobile phase consisting of 150 mM sodium phosphate (pH 7.0) containing 1.5 μM 4,4'-dianilino-1,1'-binaphthyl-5,5'-disulfonic acid (Bis-ANS; Sigma-Aldrich, St. Louis, MO, USA). Size distribution was quantified using ultraviolet (UV)-based detection with absorbance at 280 nm; intrinsic fluorescence was monitored at 340 nm after excitation at 280 nm; extrinsic fluorescence was monitored at 435 nm after excitation at 385 nm. The monomer, dimer, and aggregate peaks were quantified manually using the valleys between the peaks to define integration limits.

Peptide LC-MS/MS analysis was carried out on tryptic peptides prepared as described previously [22]. Briefly, 50 μg of each product were diluted in 6 M guanidine HCl (Sigma-Aldrich) at pH 7.8 in 25 mM ammonium bicarbonate (Sigma-Aldrich) for analysis of the protein after reduction with 10 mM dithiothreitol (Sigma, St. Louis, MO, USA) and alkylation with 30 mM iodoacetamide. Analysis of disulfides was performed on the tryptic digest after dilution of 50 μg of each IVIg product in 6 M guanidine HCl at pH 6.1 in 50 mM ammonium acetate. Free thiols were alkylated with 30 mM iodoacetamide to minimize disulfide scrambling during sample preparation. The reduced and non-reduced preparations were digested with trypsin (Trypsin Gold, Promega, Madison, WI) in a Barocyler (Pressure Biosciences, So. Easton, MA) using the conditions described previously [22]. The tryptic peptides were separated using Dionex ultra performance LC (Dionex, Sunnyvale, CA, USA) with an ethylene bridged hybrid (BEH) C18 column (Waters Corporation, Milford, MA, USA). Mass spectrometric data were acquired on a QExactive mass spectrometer (ThermoFisher Scientific, Bremen, Germany) operated in positive mode covering a mass range from 350–2000 Da. Glycosylation and isotype distribution were carried out as described previously [22]. The relative abundance of IgG4 half-antibody was quantified from the nonreduced digest based on the extracted ion area of the two most abundant charge states for both the IgG4 hinge peptide with inter-chain disulfide bonds and the IgG4 hinge peptide with an intra-chain disulfide. Allotype distribution was quantified using the peptide containing the G1m3/G1m17 determinants, while G1m1 and G1m2 allotypes were quantified using the peptide containing these allotypic determinants but quantified relative to the nG1m1 and nG1m2 isoallotypic peptides, which are also found in IgG2/3/4 [10]. Non-IgG proteins were identified using Proteome Discoverer (Thermo Fisher Scientific, San Jose, CA, USA) and searching against the full human database from UniProt (UniProt Consortium). Peptides were identified using a parent mass accuracy of 5 ppm and a fragment mass accuracy of 0.2 Da. Non-IgG proteins were quantified based on peptide spectral matches normalized to total peptide spectral matches for each sample.

SEC-MS analysis of the IVIg products after limited proteolysis, isolation of the Fab, and characterization of Fab glycosylation were carried out as described previously [22]. G1m1 and nG1m1 allotypic determinants that differ by 64 Da were quantified based on the summed intensity of all glycoforms for each allotype.

The Pearson correlations shown for allotypes were calculated in Prism6 (GraphPad, La Jolla, CA, USA) based in the relative abundance calculated for the allotypes. Both bottom-up quantitation from the peptide LC-MS analysis and middle-down quantitation from SEC-MS analysis were used for the correlation.

To analyze Fab glycosylation, the IVIg was cleaved by FabRICATOR (Genovis, Lund, Sweden or Cambridge, MA, USA) into F(ab’)_2_ and Fc fragments. IVIg was first buffer exchanged into 50 mM sodium phosphate, 150 mM sodium chloride, pH 6.6 using a Vivaspin 20 (GE Healthcare, Marlborough, MA, USA) 30 kDa MWCO device. After overnight incubation with FabRICATOR, the samples were loaded directly onto a HiTrap Protein G HP column (GE Healthcare, Marlborough, MA, USA). The column was washed with 1x PBS, 0.5 M sodium isothiocyanate, and 1x PBS. The F(ab’)_2_ containing flow-through and wash fractions were combined.

The samples were digested with PNGase F enzyme (ProZyme, Hayward, CA, USA) using reagents from the ProZyme kit. Denaturant was first added to the Fab and then heated for 10 min at 38°C. The precipitate formed was then dissolved by adding detergent. This was followed by adding deglycosylation buffer and enzyme and setting up the reaction for ~3.5 hours at 37°C in a water bath. The samples were then cleaned up using Hypercarb (Thermo Fisher Scientific) cartridges on a vacuum manifold to remove the protein pellet. The released glycans were then labeled with 2AB (2-aminobenzamide dye) for ~2.5 hours at 65°C. The last step was a cleanup of the labeled glycans to remove excess label using GlycoGlean G cartridges (ProZyme) using vacuum.

Fab glycans were analyzed by hydrophilic interaction liquid chromatography-LC-MS-fluorescence detection using an Acquity BEH Glycan column (Waters Corporation). Mass spectra were acquired on a linear trap quadropole mass spectrometer (Thermo Fisher Scientific, San Jose, CA, USA) operated in both positive and negative mode to aid in the identification of neutral and acidic species. Fab glycans were quantified based on the fluorescence signal from the 2-AB label using an excitation wavelength of 330 nm and monitoring emission at 420 nm. Co-eluting species were not quantified separately.

The nine IVIg samples were named as 1A, 1B, 1C, 2A, 2B, 2C, 3A, 3B, and 3C, and were blindly probed on HuProt™ human proteome arrays (CDI Laboratories, Mayaguez, PR, USA). The arrays were incubated with blocking buffer (5% BSA in 1x PBS plus 0.1% [v/v] Tween 20) at room temperature for 1 hour with gentle shaking, after which each of the IVIg samples was added to two HuProt™ microarrays at a final concentration of 100 μg/ml and further incubated at room temperature for 1 hour with gentle shaking. After washing three times with 1x tris-buffered saline plus 0.1% (v/v) Tween 20 (TBST) for 10 min each, the microarrays were incubated with the mixture of Alexa Fluor 647 mouse antihuman IgG Fc antibody (1:1000; BioLegend, San Diego, CA, USA) and Alexa Fluor 647 AffiniPure F(ab')_2_ Fragment Goat Anti-Human IgG, Fc fragment–specific (1:10,000; Jackson ImmunoResearch, West Grove, PA, USA) in blocking buffer in the dark at room temperature for 1 hour with gentle shaking. Subsequently, the microarrays were washed three times with TBST for 10 minutes each and then briefly rinsed three times with double-distilled water. To fully remove the double-distilled water, the microarrays were centrifuged for 3 minutes at 1000 rpm in a black microscope slide box with Kimwipes underneath. Finally, the microarrays were scanned with the GenePix 4000B Microarray Scanner (Molecular Devices, Sunnyvale, CA, USA) and the signal intensities were acquired using the GenePix Pro 7.0 software (Molecular Devices).

For the protein binding data, positive calling in each microarray was conducted according to the procedure previously described by Jeong et al [27]. Briefly, the A score (average Z score of the duplicate spots) of each probe on the HuProt™ array was calculated, and a given probe was considered positive when it’s A score was greater than 3.0. Probes that bound to the mixture of secondary antibodies alone (negative control) were excluded from further analysis. A given probe was included in the heatmap when at least one of the nine IVIg samples displayed positive signals (A score >3.0) on both of its replicate arrays. A total of 355 probes from the 18 arrays passed this threshold. Hierarchical correlation clustering was done to reveal similarities among the biological samples (columns) and the probe value (rows). Before clustering, the included A scores were first mean subtracted in order to make the resulting heatmap more comprehensible. In mean subtraction, for each probe the average A score for all 18 arrays was subtracted from each of the 18 values; as a result, the 18 new values averaged 0.0 for that probe. Because the same value, a constant, was subtracted from all the samples, correlation clustering is not affected; however, the resulting heatmap is easier to visualize and understand.

## Results

### Different IVIg products display similar size distribution but differences in extrinsic fluorescence

The specifications for IVIg in the European Pharmacopoeia require that at least 90% of the gamma globulins in IVIg be monomeric or dimeric and that no more than 3% be larger aggregates [11]. SEC is typically used for quantitation of the distribution of different MW species in IVIg.

The addition of the molecular probe Bis-ANS to the SEC mobile phase allows monitoring of the exposure of hydrophobic segments of proteins. Bis-ANS is a fluorescent dye that preferentially binds nonpolar cavities within proteins and is typically used as an indicator of protein folding because its quantum yield is highly dependent on the polarity of the environment [28]. The use of solvatochromic fluorescent probes in combination with chromatographic techniques has facilitated the detection of three-dimensional structural alterations to proteins, with increased dye fluorescence in aggregates and partially unfolded antibodies [28].

**Fig 1:**
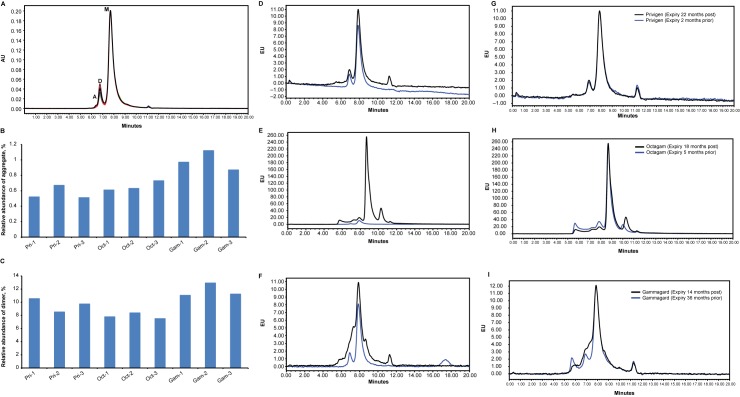
Size-exclusion chromatography analysis of 3 lots of intravenous immunoglobulin (IVIg) from 3 different manufacturers for a comparison of 9 different IVIg materials. (A) UV 280 nm detection; peak A, aggregate; peak D, dimer; peak M, monomeric IgG. (B) Abundance of aggregate (peak A, panel A). (C) Abundance of dimer (peak D, panel A). Overlay of intrinsic fluorescence (excitation 280 nm, emission 340 nm; blue) and extrinsic (Bis-ANS) fluorescence (excitation 385 nm, emission 435 nm; black) for (D) Privigen, (E) Octagam, and (F) Gammagard. Overlay of extrinsic fluorescence for fresh (back) and expired (blue) lots of (G) Privigen, (H) Octagam, and (I) Gammagard. Single lots of each product were analyzed prior to or after the expiration date: Pri-1 (22 months after), Pri-2 (2 months prior), and Pri-3 (28 months after); Oct-1 (5 months prior), Oct-2 (12 months after), and Oct-3 (24 months after); Gam-1 (6 months after), Gam-2 (36 months prior), and Gam-3 (24 months after). Gam, Gammagard lot; Oct, Octagam lot; Pri, Privigen lot.

The UV chromatograms for nine different IVIg samples (3 separate lots of each of the 3 products) are shown in Fig 1A. The size distribution was largely similar for all nine IVIg samples, which each met the specifications set by the European Pharmacopoeia [11]. In this analysis, Gammagard contained slightly higher levels of both dimer and larger aggregates compared with Privigen and Octagam (Fig 1B and 1C).

However, each product displayed characteristic intrinsic and extrinsic fluorescence profiles. Representative intrinsic fluorescence profiles due to tryptophan residues (excitation 280 nm, emission 340 nm) and the extrinsic fluorescence profiles due to the dye (excitation 385 nm, emission 435 nm) for the three products are shown in Fig 1D–1F. The extrinsic fluorescence of Privigen largely mirrored the intrinsic fluorescence, both in intensity and profile, suggesting that it contained low levels of partially unfolded IgG and nonnative aggregates. The profile of Octagam showed the presence of a low MW species as indicated by strong fluorescence from the dye. The intensity of the extrinsic fluorescence for the low MW peak was increased 10-fold compared to this product’s IgG monomer peaks. The species giving rise to this fluorescence was not detected in the UV or intrinsic fluorescence signals, so the abundance cannot be determined; however, its presence suggested a qualitative difference between the products. In addition, the extrinsic fluorescence profile of Octagam revealed the presence of larger MW species not seen by UV absorbance or intrinsic fluorescence, suggesting the low MW species was present at low abundance. The extrinsic fluorescence profile of Gammagard suggested the presence of a species eluting between the monomer and dimer peaks. Because SEC separates on the basis of hydrodynamic volume, this intermediate species may represent misfolded monomer. The presence of the misfolded Bis-ANS binding species in some IVIg products may be a concern for the formation of aggregates according to the generally accepted models of protein aggregation, in which the native state (N) is in equilibrium with a perturbed state (U) from which aggregates (A) are irreversibly formed.

N↔U→A

To gain a greater understanding of the Bis-ANS–complexed species, we also analyzed IVIg products after their expiration date (Fig 1G–1I). Lots of Privigen IVIg analyzed before and after their expiration dates displayed similar extrinsic fluorescence profiles (Fig 1G), and Octagam (Fig 1H) showed only minor changes. The extrinsic fluorescence profile of an expired lot of Gammagard showed a decrease in the potential misfolded monomer and an increase in larger aggregate species (Fig 1I).

### Differences in undesired modifications are observed in different products

**Fig 2:**
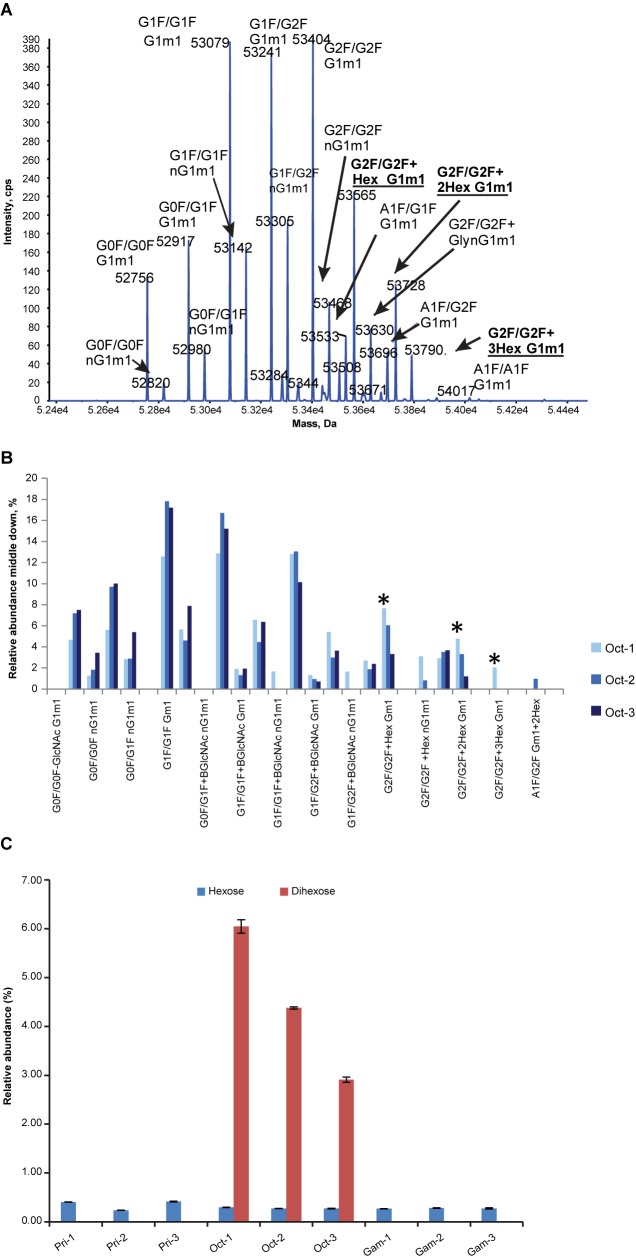
Nonenzymatic glycation analysis of Octagam lots. (A) Deconvoluted mass spectrum of papain cleaved Fc from Oct-1 (expired 5 months prior to analysis). Highlighted glycoforms labeled with “Hex,” “2Hex,” or “3Hex” indicate modified glycoforms with additional hexose units. (B) Relative quantitation of immunoglobulin G1 Fc glycoforms analysis of papain cleaved Fc from three lots of Octagam showing product age–related glycation; Oct-1 (5 months prior to expiration), Oct-2 (12 months after expiration), and Oct-3 (24 months after expiration). (C) Peptide liquid chromatography–mass spectrometry/mass spectrometry relative quantitation of nonenzymatic glycation of the peptide VSNK*ALPAPIEK from the CH2 domain of immunoglobulin G1. Data in panel C are displayed as mean ± standard deviation (n = 3 technical replicates). *Denotes glycated species. Gam, Gammagard lot; Oct, Octagam lot; Pri, Privigen lot.

Optimal protein formulations are critical to maximize the solubility and stability of protein therapeutics. However, some protein formulation components may lead to undesirable modifications to the active ingredient. For example, formulations containing reducing sugars have the potential to generate glycation of the protein [29]. Protein glycation is an undesired modification given its potential to generate advanced glycation end products, which have been associated with negative effects on human health [30,31]. Octagam is formulated using maltose (a reducing sugar) as a stabilizer, whereas Privigen and Gammagard use proline and glycine, respectively [4,6,10]. Three lots of Octagam with expiration dates ranging from 5 months before analysis to 2 years after analysis were analyzed. Middle-down analysis of the IgG1 isotype from these lots by SEC-ToF-MS showed the presence of glycated IgG1 Fc domain (Fig 2A).

Quantitation from the middle-down analysis shows age-related differences in levels of glycation for the Octagam lots (Fig 2B). Site-specific nonenzymatic glycation of a single solvent-exposed lysine residue was compared across lots and between products. Modification of this residue by a single hexose residue was similar between all three products as shown by peptide LC-MS/MS (Fig 2C). However, modification of this residue by the addition of maltose, a dihexose, was seen exclusively in Octagam, suggesting that this modification was a result of maltose in the formulation. The abundance of this modified lysine followed the same trend seen in the middle-down analysis in that older material had higher levels of the modified residue. Substantial differences were seen when comparing two lots that had not yet reached expiration date at the time of analysis.

### Abundance of IgG3 and IgG4 and the characteristics of IgG4 vary between IVIg products

**Fig 3:**
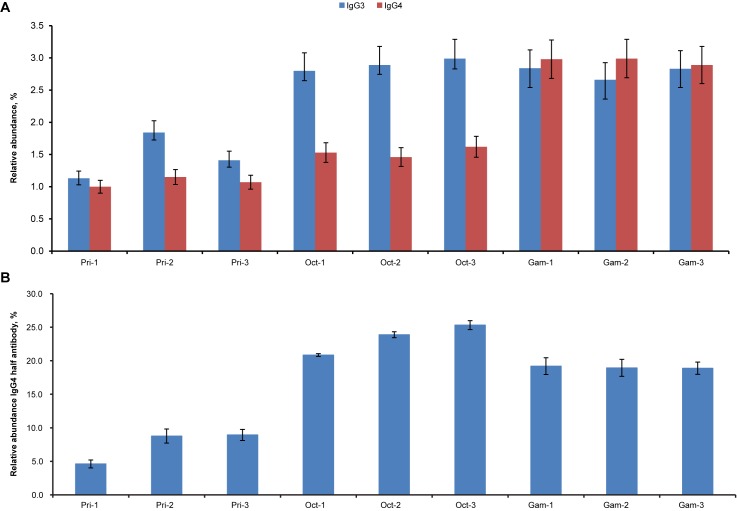
**Relative abundance of IgG3 and IgG4 isotypes (A) and IgG4 half antibody (B) in different lots of intravenous immunoglobulin products.** Data are displayed as mean ± standard deviation (n = 3 technical replicates). Single lots of each product were analyzed prior to or after the expiration date: Pri-1 (22 months after), Pri-2 (2 months prior), and Pri-3 (28 months after); Oct-1 (5 months prior), Oct-2 (12 months after), and Oct-3 (24 months after); Gam-1 (6 months after), Gam-2 (36 months prior), and Gam-3 (24 months after). Gam, Gammagard lot; Oct, Octagam lot; Pri, Privigen lot.

Given the differences in the interaction of each IgG isotype to Fc_γ_-receptors [14], it may be important to maintain a specific distribution of each isotype in the IVIg products. The physiological ranges of serum IgG3 and IgG4 are 1.7%-4.5% and 0.8%-11.7%, respectively [32]. The isotype distribution of IVIg should largely mirror the distribution in normal human serum. However, subphysiological levels of IgG3 and IgG4 have been reported in certain IVIg products [33]. A comparison of the low abundance isotypes IgG3 and IgG4 between the products investigated in the current analysis revealed subtle differences (Fig 3A). The levels of IgG3 varied from 1%–2% in Privigen and from 2.5%–3% in Gammagard and Octagam. IgG4 levels range from approximately 1% of total IgG in Privigen to approximately 3% in Gammagard. The ratio of IgG3/IgG4 varied between the products from approximately 1:1 in Privigen and Gammagard to approximately 2:1 in Octagam.

IgG4 antibodies are unique in that they form stable half antibodies and undergo dynamic Fab arm exchange [17]. Octagam and Gammagard each contain a high percentage of IgG4 with intra-chain disulfides (approximately 19%–25%; Fig 3B) as has been reported in serum [34]. However, Privigen had a low fraction of IgG4 half antibody (approximately 5%–9%) (Fig 3B).

### IgG1 allotype distribution differs between products and between different lots of Privigen

The description of the allotypes in human immunoglobulins and their potential effects on immunogenicity in therapeutic products has been previously reported [16]. Quantitation of the allotype distribution may provide information about the source plasma for the three products tested. Three allotypic variants were quantified for each product by peptide LC-MS/MS. The G1m17/G1m3 variant is found in the CH1 domain and consists of a lysine-to-arginine substitution. The G1m1/nG1m1 variant is found in the CH3 domain, and consists of aspartic acid to glutamic acid and leucine to methionine substitutions. The G1m2/nG1m2 variant is also found in the CH3 domain and consists of a glycine to alanine substitution.

**Fig 4:**
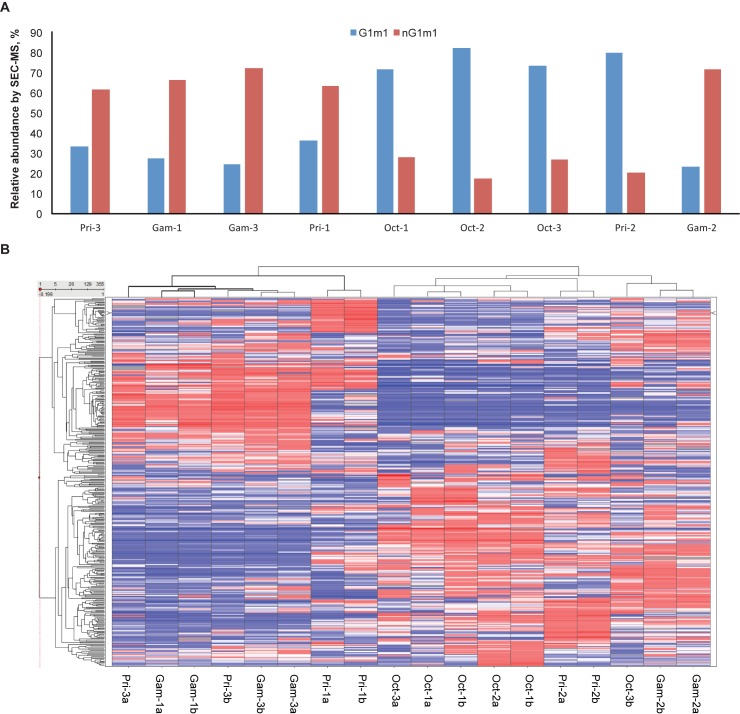
**Allotype distribution for the G1m1/nG1m1 isoallotypes by size-exclusion chromatography–time of flight–mass spectrometry (A) and heatmap of positive probes bound by intravenous immunoglobulin (IVIg) products on HuProt™ human proteome arrays (B).** A total of 355 proteins on the HuProt™ arrays showed positive binding signals to the nine IVIg samples. These positive probes were clustered in the heatmap according to their A scores reflecting the signal intensities. Red color shows that a given sample has a higher-than-average A score for the given probe, and blue color reflects a lower-than-average A score. Each IVIg sample was probed on 2 arrays labeled as “–#a” and “–#b,” respectively. Single lots of each product were analyzed prior to or after the expiration date: Pri-1 (22 months after), Pri-2 (2 months prior), and Pri-3 (28 months after); Oct-1 (5 months prior), Oct-2 (12 months after), and Oct-3 (24 months after); Gam-1 (6 months after), Gam-2 (36 months prior), and Gam-3 (24 months after). Gam, Gammagard lot; Oct, Octagam lot; Pri, Privigen lot.

Allotype distribution varied between the different products and within separate lots of some products. Relative quantitation of the G1m1/nG1m1 by middle-down SEC-MS showed that Octagam tended to have higher levels of the G1m1 determinant and lower levels the of nG1m1 determinant compared to Gammagard, which contained lower levels of the G1m1 determinant and higher levels of the nG1m1 determinant (Fig 4A). Privigen presented an interesting case because the allotype distribution is dependent on the lot of material and is potentially related to the date of manufacture. In this product, two of the three lots had an allotypic signature similar to Gammagard while the other lot was similar to Octagam (Fig 4A). Quantitation of the other allotypic determinants by bottom-up peptide LC-MS showed a similar trend. There were higher levels of the G1m2 and G1m3 determinants in all three lots of Gammagard and Privigen lots Pri-1 and Pri-3 compared with Octagam and Privigen lot Pri-2, which contained higher levels of the G1m1 determinant (Figs A-C in S1 Fig). The three allotypic sites in IgG1 correlated with one another based on the Pearson correlation between the G1m3 and nG1m1 allotypes (r = 0.9654) and between the G1m2 and G1m1 allotypes (r = –0.9703) (Fig D and Fig E in S1 Fig). It is interesting to note that based on their expiration dates, Pri-1 and Pri-3 were produced at similar times whereas Pri-2 was produced substantially earlier.

### Human protein array binding analysis reveal differences in immunoglobulin antigen binding repertoire

IVIg is composed of a wide repertoire of antibodies with different specificities. To profile the distribution of antibody specificities in different IVIg products we used a human proteome (HuProt™) microarray containing more than 19,000 human proteins. Each of the nine IVIg samples were blindly probed on two HuProt™ arrays to ensure reproducibility. Positive binding signals from the different IVIg products were identified on 355 proteins on the array. Using clustering analysis, a heatmap of the 355 proteins was generated based on the binding signal intensities of each of the IVIg products (Fig 4B). The products clustered primarily into two different groups according to the pattern of positive hits for each sample. Based on this blinded analysis, the three lots of Octagam clustered within the same group (right side of the heatmap) while two lots of Gammagard (Gam-1 and Gam-3) clustered together in the other main group (left side of the heatmap). The Gam-2 lot did not cluster with the other Gammagard lots. It is important to note, however, that the Gam-2 lot is the only lot that expired years before the analysis took place. We also noticed that two lots of Privigen (Pri-1 and Pri-3) clustered in with Gam-1 and Gam-3 (left side of the heatmap), while the other Privigen lot (Pri-2) clustered with the Octagam lots (right side of the heatmap). These results suggested that the Pri-2 antibodies could share more common characteristics with the Octagam lots, while Pri-1 and Pri-3 share more common characteristics with Gam-1 and Gam-3. Of the human proteins probed on the array, IVIg bound most strongly to cytoplasmic and nuclear protein; this is consistent with data as published by Bussone et al [35], Néron and Roy [36], and Wymann et al [37]. The specific proteins bound differentially between cluster 1 containing Gam-1, Gam-3, Pri-1, and Pri-3 and cluster 2 containing Oct-1, Oct-2, Oct-3, and Pri-2 were examined. Twelve proteins with A-scores greater than 3 for all samples and had at least a 2-fold change in modified A-score had significantly different reactivity (P<0.01) when compared between the two clusters. Of the 12 proteins that fit this criteria, eight are cytoplasmic or nuclear proteins. The Octagam cluster had higher reactivity to two membrane proteins: SLAMF6 and SLC16A4 (**S1 Table**).

### Fab and isotype-specific Fc glycosylation

**Fig 5:**
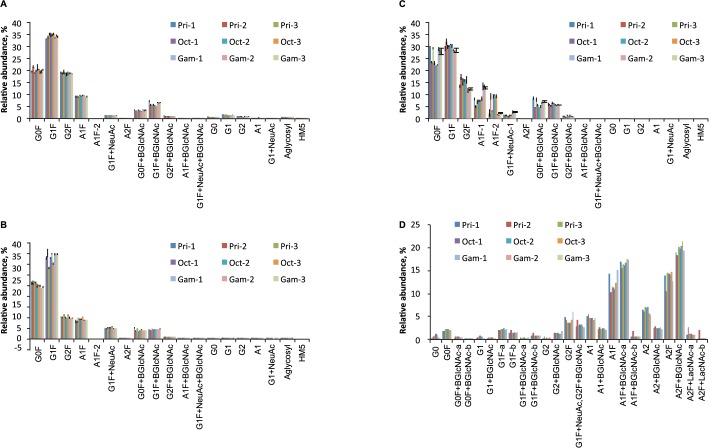
**Fc glycosylation of IgG1 (A), IgG2/3 (B), IgG4 (C) and Fab (D) for different intravenous immunoglobulin products.** Data are displayed as mean ± standard deviation (n = 3 technical replicates). Single lots of each product were analyzed prior to or after the expiration date: Pri-1 (22 months after), Pri-2 (2 months prior), and Pri-3 (28 months after); Oct-1 (5 months prior), Oct-2 (12 months after), and Oct-3 (24 months after); Gam-1 (6 months after), Gam-2 (36 months prior), and Gam-3 (24 months after). Gam, Gammagard lot; Oct, Octagam lot; Pri, Privigen lot.

N-glycosylation in the Fc domain of IgG1 (Fig 5A) and IgG2/3 (Fig 5B) was similar for all three products. Slight enrichment of glycans G0F and G1F with bisecting N-acetylglucosamine was seen for IgG1 in Gammagard and for a single lot of Privigen (Fig 5A). Lot-to-lot variations in the levels of G1F on IgG2/3 were seen in Privigen and Octagam (Fig 5B).

More substantial differences were seen in the glycosylation of IgG4 (Fig 5C). Levels of the agalactosyl G0F glycopeptide were higher in all three lots of Gammagard compared with Octagam. Levels of agalactosyl glycans in Privigen appeared to be dependent on the lot. Privigen lots Pri-1 and Pri-3 had “Gammagard-like” galactosylation, whereas Privigen lot Pri-2 had “Octagam-like” galactosylation. Overall, Octagam had higher sialylation and Privigen had lower sialylation of the IgG4 isotype. The levels of sialylation in Privigen appeared to depend on the lot, with higher levels observed in lot Pri-2 (Fig 5C). In these lots of IVIg, IgG4 glycosylation appeared to be associated with IgG allotype based on the similar patterns observed between these two attributes among lots and products.

Fab glycosylation varied by lot but not substantially among products. Glycosylation of the Fab domain was characterized by higher levels of sialylation and bisecting N-acetylglucosamine and by lower levels of fucosylation compared to the Fc; this was consistent with recent reports [38]. The glycosylation of Privigen lot Pri-2 and Gammagard lot Gam-3 was slightly different from other lots and characterized by a slightly lower level of bisecting N-acetylglucosamine (Fig 5D).

### Comparison of non-IgG background proteins

**Fig 6:**
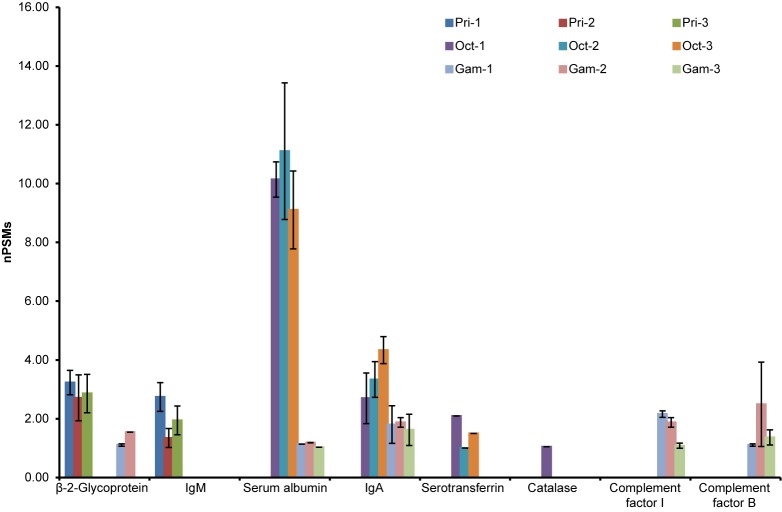
Mass spectrometry proteomics analysis of non–IgG proteins in intravenous immunoglobulin products. Data are displayed as mean ± standard deviation (n = 3 technical replicates). Single lots of each product were analyzed prior to or after the expiration date: Pri-1 (22 months after), Pri-2 (2 months prior), and Pri-3 (28 months after); Oct-1 (5 months prior), Oct-2 (12 months after), and Oct-3 (24 months after); Gam-1 (6 months after), Gam-2 (36 months prior), and Gam-3 (24 months after). Gam, Gammagard lot; nPSMs, normalized peptide spectral matches; Oct, Octagam lot; Pri, Privigen lot.

IVIg contains multiple human proteins in addition to immunoglobulins. The abundance of these proteins in the final product is largely driven by the process used to isolate the IgG fraction. Because there is not a standardized procedure for this isolation it is expected that the background proteome will vary between products. To compare the relative levels of non-IgG proteins in the different IVIg products, mass spectrometry proteomics analysis was performed on each lot of the IVIg products. Each of the products tested in the current analysis was found to have a unique distribution of high abundance background proteins (Fig 6). Privigen was characterized by higher levels of β-2-glycoprotein and IgM compared with Octagam and Gammagard. Compared with the other products, Octagam was found to have elevated levels of IgA, serotransferrin, and, in particular, serum albumin, which was the most abundant non-IgG protein detected in any of the products. Gammagard had relatively high levels of complement proteins compared with the other products, specifically factor I and factor B. The peptides identified for each of the non-IgG proteins and the number of peptide spectral matches are shown in S2 Table.

## Conclusions

The mechanism of action of IVIg in autoimmune and inflammatory conditions remains a subject of significant debate [13]. The anti-inflammatory activity of IVIg has been proposed to be associated with multiple different components of the mixture; therefore, physicochemical characterization of different IVIg products and within-product lots may provide insight into differences that could potentially affect a patient’s experience [11]. For example, the IgA content in certain products has been associated with anaphylaxis in IgA-deficient patients receiving IVIg [39]. The content of dimers and aggregates in IVIg are also associated with activity and safety [13].

In this study, high-resolution analytics were applied to characterize three approved IVIg products—Gammagard, Privigen, and Octagam—and identify differences in the distribution of MW species, IgG sequence variants, isoforms, glycoforms, and the repertoire of antibody-binding specificities. Although the concentration and the formulation may have an impact on the stability of the drug products, it is unlikely that the concentration would have a substantial impact on the analytical results. Prior to analysis the drug products were diluted a minimum of 5-fold into the same solvent to minimize any differences. The effect of aging on these products was also examined to identify product-specific changes in size distribution, and post-translational modification profiles. Some of the differences identified related to mechanisms reported to influence the activity of IVIg and the stability of protein therapeutics. For example, high MW species were detected in both Octagam and Gammagard but not in Privigen. The abundance of these species appeared to increase as a function of the age of the product. These low abundance species are only apparent in the Bis-ANS profile, which has high sensitivity for aggregates [28]. Despite this low abundance, the aggregate fraction of IVIg has been proposed to have increased potency as a competitive inhibitor of Fc_γ_ receptor–mediated immune cell activation by pathogenic immune complexes [40]. More importantly, aggregates also have immunogenic potential that increases with aggregate size [41]. However, the extent to which any of the differences observed could impact the safety or efficacy profile of the drug is unknown and may warrant further investigation.

The isotype distribution varied among different IVIg products; levels of IgG3 and IgG4 in particular were most variable. Because each IgG isotype is known to interact with different affinities with Fcγ receptors, differences in the isotype distribution may influence efficacy of the drug in certain indications [14]. Deeper characterization of the IgG4 isotype also revealed substantially lower levels of the half-antibody in all three lots of Privigen. These half-antibodies represent the first step in dynamic Fab exchange, which is a hallmark of IgG4 and has been proposed to give IgG4 anti-inflammatory activity [17]

The importance of glycosylation to antibody activity has been well studied in recent years. Glycosylation of the Fc domain has been reported to drive certain aspects of IVIg activity [20,42]. Sialylation of the Fc domain has been shown to play an important role in the potency of IVIg in multiple animal models of inflammation [22]. In this comparison, the glycosylation pattern did not vary substantially between the different presentations, particularly for IgG1 and IgG2/3. Minor differences in the abundance of sialylated species were seen in the IgG4 molecule. However, the role of different IgG isotypes in the sialylation-mediated anti-inflammatory activity of IVIg remains unclear.

The comparison of the allotype distribution of IgG1 among the different products indicates that all manufacturers used plasma sourced from different populations because IgG allotypes are not equally distributed across human populations. All IVIg products tested contained a mixture of IgG1 allotypes, although at different ratios. The G1m1 allotype, for example, accounted for 15%–80% of IgG1 molecules across the different products and lots tested. Furthermore, different lots of Privigen also appeared to source plasma from different populations.

The formation of anti-allotypic antibodies has been examined for mAbs. Two small studies failed to demonstrate a link between allotypic mismatch and antidrug antibody formation with chimeric [43] and fully human anti-tumor necrosis factor antibodies [44]. These results suggest allotype distribution may not be important in of itself.

It is interesting to note that the pattern of antigen binding on the HuProt™ array appeared to be associated with the allotype distribution. This suggests that like allotype distribution, the antigen binding profile of IVIg may reflect the population from which IVIg is drawn. The differences between lots of Privigen may also relate to manufacture date, as the Pri-1 and Pri-3 lots had expiration dates 22 and 28 months after the analysis was performed. The Pri-2 lot, on the other hand, expired 2 months prior to the analysis.

Octagam is formulated in the presence of maltose, a reducing sugar [3]. The characterization of multiple lots of Octagam showed increasing levels of nonenzymatic glycation as a function of product age. The highest levels were seen in an expired lot; furthermore, a twofold difference in levels of glycation was seen in two separate lots within their expiration dates. Previous studies on the effect of nonenzymatic glycation of IgG suggest that this modification does not influence the affinity of the Fc for Fc_γ_RIIIa or FcRn [45]. However, glycation has been reported to influence the stability of proteins and can induce amyloid fibril formation in proteins such as albumin [46], which we found at relatively high abundance in Octagam.

Non-IgG proteins in IVIg have been associated with adverse events; the best documented example is the association of FXIa with thromboembolism in recalled lots of Octagam [47]. β2-glycoprotein, one of the proteins identified as slightly elevated in Privigen in this study, has also been discussed as a potential risk factor for thromboembolism [47,48]. An article by Sridhar et al examined literature from 2008 to 2012 that detailed adverse events associated with IVIg use [49]. Their analysis showed a nonsignificant enrichment of thromboembolic events in Octagam and Privigen compared with Gammagard Liquid [49]. None of the lots of material analyzed herein were associated with thromboembolic events based on the recalled lot numbers from 2010. It is important to note that the lots of Privigen analyzed in this manuscript expired between 2014 and 2016. Based on the shelf life of Privigen (36 months, beginning in 2010), only one of the lots could have been in use during the time period covered in the retrospective study, during which time thromboembolic events were not elevated in Privigen. Therefore, drawing a direct line between the β2-glycoprotein levels found in Privigen and any treatment-emergent serious adverse events is beyond the scope of this manuscript.

In summary, this high-resolution comparison of the physicochemical properties of different IVIg products has identified differences in components of the mixture across the different products and within lots of the same product. The data presented in this paper highlight product-specific differences in size distribution, stability, allotype distribution, antigen binding profile, and background proteins. In addition to product-specific differences, lot-to-lot differences in allotype distribution were also demonstrated for one of the products (Privigen), suggesting that different lots may come from different donor populations. Awareness of differences in physicochemical attributes may ultimately lead to optimization of IVIg products for treatment of autoimmune diseases.

## Supporting information

S1 Fig **Allotype distribution of the G1m1 (A), G1m2 (B), and G1m3 (C) allotypes by peptide LC-MS/MS.** The plot of abundance of G1m3 allotype was measured by peptide LC-MS/MS vs abundance of nG1m1 isoallotype measured by middle-down SEC-MS, and (D) G1m2 isoallotype was measured by peptide LC-MS/MS vs G1m1 allotype measured by middle-down SEC-MS (E). Data in panels A–C are displayed as mean ± standard deviation (n = 3 technical replicates). Gam, Gammagard lot; Oct, Octagam lot; Pri, Privigen lot; r, Pearson correlation coefficient.
(PDF)

S1 Table Specific proteins bound differentially between cluster 1 containing Gam-1, Gam-3, Pri-1, and Pri-3 and cluster 2 containing Oct-1, Oct-2, Oct-3, and Pri-2. Proteins with A-score >3 for all samples and significantly different reactivity (P< 0.01; fold change >2) between the clusters.
(PDF)

S2 Table Peptides identified from high-abundance background proteins. PSM, peptide spectral matches; PSM#, peptide spectral matches count.
(PDF)
