# Supplementary figures and images for: High-resolution physicochemical characterization of different intravenous immunoglobulin products
Source: PLoS One. 2017 Jul 31;12(7):e0181251. doi: 10.1371/journal.pone.0181251 (PMC5536303; doi:10.1371/journal.pone.0181251)

S1 Fig.

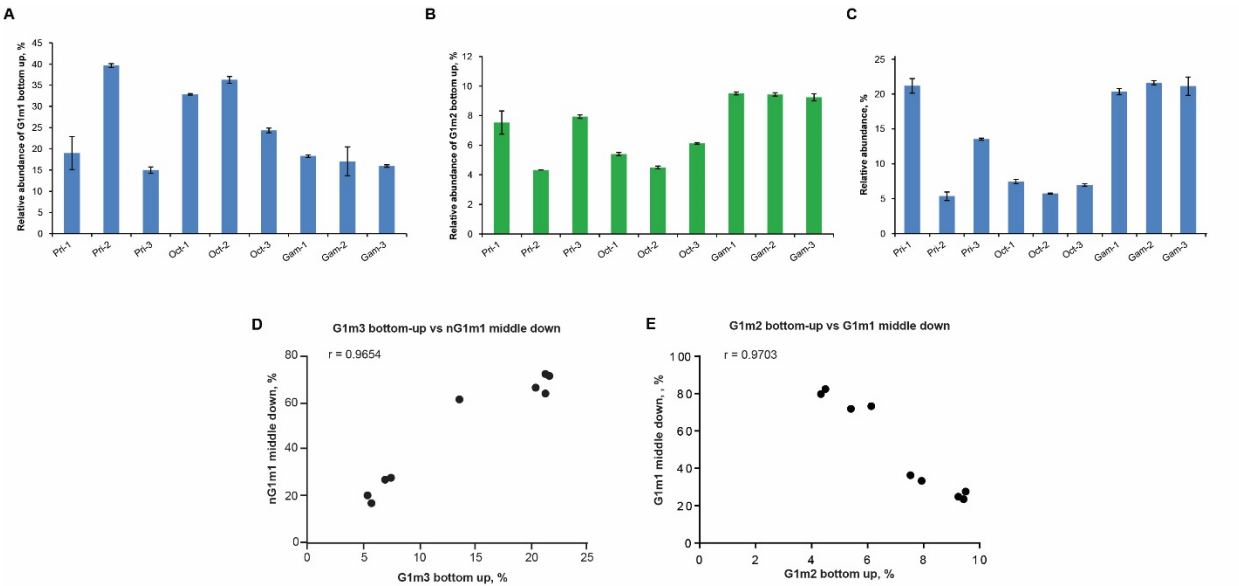

Supplement: S1 Fig — Allotype distribution of the G1m1 (A), G1m2 (B), and G1m3 (C) allotypes by peptide LC-MS/MS. The plot of abundance of G1m3 allotype was measured by peptide LC-MS/MS vs abundance of nG1m1 isoallotype measured by middle-down SEC-MS, and (D) G1m2 isoallotype was measured by peptide LC-MS/MS vs G1m1 allotype measured by middle-down SEC-MS (E). Data in panels A–C are displayed as mean ± standard deviation (n = 3 technical replicates). Gam, Gammagard lot; Oct, Octagam lot; Pri, Privigen lot; r, Pearson correlation coefficient. (PDF) [file pone.0181251.s001.pdf]
