# Supplementary material for: High-resolution physicochemical characterization of different intravenous immunoglobulin products
Source: PLoS One. 2017 Jul 31;12(7):e0181251. doi: 10.1371/journal.pone.0181251 (PMC5536303; doi:10.1371/journal.pone.0181251)
Supplement: S1 Table — Proteins with A-score >3 for all samples and significantly different reactivity (P< 0.01; fold change >2) between the clusters. (PDF) [file pone.0181251.s002.pdf]

S1 Table.

**Higher Octagam cluster**			
Gene symbol	Fold change	Location	Function
RBPJ	3.5	Nucleus/cytoplasm	Transcriptional regulator
SLC16A4	2.7	Membrane	Monocarboxylate transporter
SLAMF6	2.3	Membrane	Regulator of NK cell activation
HDGFL1	2.3	Nucleus/cytoplasm	Unknown may be involved in DNA binding
RWDD1	2.1	Cytoplasm	Unknown may protect DRG2 from proteolysis
ABI3	2.0	Cytoplasm	Actin binding
**Higher in Gammagard cluster**			
Gene symbol	Fold change	Location	Function
MARCKSL1	2.1	Cytoplasm/membrane	Actin skeleton rearrangement
VCX3A	2.4	Nucleus	Spermatogenesis
PTGES3L-AARSD1	2.4	Unknown	Conjoined gene
PGK2	2.6	Cytoplasm	Spermatogenesis
NFKB2	2.7	Nucleus/cytoplasm	Transcription factor
ANXA6	3.3	Cytoplasm	Calcium dep phospholipid binding
