# Supplementary material for: High-resolution physicochemical characterization of different intravenous immunoglobulin products
Source: PLoS One. 2017 Jul 31;12(7):e0181251. doi: 10.1371/journal.pone.0181251 (PMC5536303; doi:10.1371/journal.pone.0181251)
Supplement: S2 Table — PSM, peptide spectral matches; PSM#, peptide spectral matches count. (PDF) [file pone.0181251.s003.pdf]

S2 Table.

**Serum albumin**	**PSMs**	**Ig alpha-1 chain C region**	**PSM#**	**Complement factor I**	**PSM#**
RHPDYSVVLLLR	12	QEPSQGTTTFAVTSILR	20	HGNTDSEGIVEVK	9
VFDEFKPLVEEPQNLIK	13	TPLTATLSK	16	VFSLQWGEVK	10
KQTALVELVK	10	SAVQGPPER	12	AQLGDLPWQVAIK	8
HPYFYAPELLFFAK	13	TFTCTAAYPESK	5	YQIWTTVVDWIHPDLK	2
RHPYFYAPELLFFAK	13	WLQGSQELPR	3	IIFHENYNAGTYQNDIALIEMK	2
DVFLGMFLYEYAR	11	DASGVTFTWTPSSGK	3	**Ig mu chain C**	**PSM#**
LDELRDEGK	7	VAAEDWK	9	QVGSGVTTDQVQAEAK	4
LVNEVTEFAK	12	YLTWASR	7	QIQVSWLR	4
FKDLGEENFK	12	**Complement factor B**	**PSM#**	ESGPTTYK	4
QTALVELVK	11	DFHINLFQVLPWLK	1	VSVFVPPR	4
AEFAEVSK	12	VSEADSSNADWVTK	4	DGFFGNPR	3
LVTDLTK	11	EELLPAQDIK	5	**Beta-2-glycoprotein 1**	**PSM#**
FQNALLVR	3	CLVNLIEK	1	ATVVYQGER	13
YLYEIAR	5	EAGIPEFYDYDVALIK	6	FKNGMLHGDK	10

KVPQVSTPTLVEVSR	14	DLEIEVVLFHPNYNINGK	3	KATVVYQGER	4
VPQVSTPTLVEVSR	4	DISEVVTPR	6	NGMLHGDK	1
DAHKSEVAHR	3	ALFVSEEEKK	1	EHSSLAFWK	12
DDNPNLPR	6	DAQYAPGYDK	3	**Serotransferrin**	**PSM#**
ALVLIAFAQYLQQCPFEDHVK	1	GTDYHKQPWQAK	1	YLGEEYVK	5
LVAASQAALGL	1	**Catalase**	**PSM#**	SKEFQLFSSPHGK	4
DLGEENFK	3	GPLLVQDVVFTDEMAHFDR	2	EGYYGYTGAFR	6
KYLYEIAR	1	FNTANDDNVTQVR	2		
